# Near-Infrared Spectroscopy Reveals Abnormal Hemodynamics in the Left Dorsolateral Prefrontal Cortex of Menopausal Depression Patients

**DOI:** 10.1155/2017/1695930

**Published:** 2017-02-14

**Authors:** Xiang-Yun Ma, Yong-Jun Wang, Bo Xu, Kun Feng, Gao-Xiang Sun, Xiao-Qian Zhang, Xiao-Min Liu, Chen-Yu Shen, Xia-Jin Ren, Jing-Jing Sun, Po-Zi Liu

**Affiliations:** ^1^Medical Center, Tsinghua University, Beijing, China; ^2^YuQuan Hospital, Tsinghua University, Beijing, China; ^3^Tianjin Anding Hospital, Tianjin, China

## Abstract

*Background/Objective*. Menopausal depression (MD) is characterized by depressive symptoms along with hormonal fluctuations. We investigate brain function alteration between major depressive disorder (MDD) and MD.* Methods*. The difference in oxygenated hemoglobin (Oxy-Hb) for the prefrontal cortex (PFC) was compared retrospectively among 90 females presented with 30 MDD, 30 MD, and 30 healthy controls (HCs) using verbal fluency task (VFT) with near-infrared spectroscopy (NIRS).* Results*. We observed a significant difference in Oxy-Hb alteration in the left dorsolateral PFC (DLPFC) using VFT with NIRS (channel 18, *P* = 0.007) between the MD and MDD groups. A significant difference in Oxy-Hb levels was observed among the three groups in the bilateral DLPFC (channels 18, 27, 33, 39, 41, and 45; *P* < 0.05). Compared to the HCs, the MD group presented lower Oxy-Hb activation in the right DLPFC (channel 41; *P* = 0.048) and the left DLPFC (channels 18, 39, and 45; *P* < 0.05), and the MDD group presented lower Oxy-Hb activation in the right DLPFC (channels 27, 33, and 41; *P* < 0.05) and the left DLPFC (channels 39 and 45; *P* < 0.05).* Conclusion*. Abnormal hemodynamics of the left DLPFC can differentiate MD from MDD by NIRS.

## 1. Introduction

Major depressive disorder (MDD) is a severe mental disorder that affects both males and females and has a lifetime prevalence of 6.7% in the general population [1]. It is characterized by a change in mood accompanied by psychophysiological manifestations such as loss of appetite, lack of sleep or sexual desire, and lower self-esteem. The prevalence of MDD is different between males and females; data from the National Health and Nutrition Examination Survey show that females have a higher tendency to develop depression compared to males (6.7% versus 4.0%, resp.) [2]. Furthermore, at the beginning of the 21st century, the prevalence of MDD was 9.5% for females, demonstrating that females are more likely to develop MDD than males [3, 4].

In females, data from the National Health and Nutrition Examination Survey show that the depression rates in those aged 40–59 years old are higher than any other age group [2]. This finding is attributed to the hormonal changes that occur during the premenopausal period. Menopause is a biopsychosocial phenomenon defined as a stage in the process of female aging rather than an event, which generally occurs in those older than 40 years old [5, 6]. During this period, females experience fluctuating hormonal levels like decreased amounts of estrogen and increased levels of follicle-stimulating hormone, which result in significant changes to the rhythm of their menstrual cycle [7]. It is also accompanied by vasomotor symptoms like hot flushes, insomnia, and altered sexual desire. Therefore, females become more vulnerable to psychological symptoms including depression, anxiety, irritability, and tearfulness. Previous research has associated the menopausal stage with an increased risk of major depression [8]. Furthermore, studies have reported that the fluctuating levels of estrogen are related to a depressed mood in middle-aged females [9, 10]. In this study, we refer to depression that occurs during the menopausal transition as menopausal depression (MD).

Hormonal fluctuation might be linked to altering the structure of the prefrontal cortex and hippocampus and producing declines in the memory and processing speed as well as executive function [11]. Moreover, Wang et al. have suggested that estrogen might adjust the white matter microstructure of the insula and subcortical regions [12]. Finally, Hara et al. have proposed that estrogen can affect the prefrontal cortex and hippocampus by inducing spinogenesis and synaptogenesis [13].

Abnormal neuroimaging results have been reported previously in cases presented with MDD [14]. In addition, approximately 80% of depression patients have reported some degree of functional impairment in the National Health and Nutrition Examination Survey [2]. Additionally, certain neuroimaging techniques, such as magnetic resonance imaging, positron emission tomography, and near-infrared reflectance spectroscopy (NIRS), have demonstrated the existence of functional changes and altered structures in the human brain during MDD [15–17].

NIRS is a spectroscopic technique that uses light sources within the spectral window from 650 to 1000 nm and it can penetrate biological tissues that have a density between those of water and hemoglobin [18]. Due to the noninvasive nature of the technique and the portability of the spectroscopic scanner, NIRS has been applied to many medical fields such as cognition, psychiatric disorders, and preoperative functional assessment [19]. NIRS can detect changes in the oxygenation levels of hemoglobin. Cortical activation is usually reflected by an increase of local cerebral blood flow and cerebral blood volume, which lead to an increase of oxygenated hemoglobin (Oxy-Hb) and a decrease in deoxygenated hemoglobin (HHb) [20]. Among a number of psychiatric disorders, NIRS has been applied to MDD, schizophrenia, bipolar disorder, and other psychiatric disorders [21].

Previous studies have emphasized the critical role of the prefrontal cortex (PFC) in the pathology of depression through its role in cognition, executive, and emotional functions [22–24]. However, differences in the PFC functions in MD and MDD have not been the subject of major research yet. Therefore, in this study, we investigated the change of brain function, specifically in the PFC, among healthy controls (HCs) and subjects with MD or MDD using a verbal fluency task (VFT) with NIRS.

## 2. Materials and Methods

### 2.1. Ethics Statement

The protocol for this study was reviewed and approved by the Ethics Committee of YuQuan Hospital, Tsinghua University, Beijing, China. All participants signed an informed written consent prior to enrollment.

### 2.2. Participants

This was a retrospective study in which a total of 90 female participants were recruited from July 2013 until July 2015 from the Psychiatry Department of YuQuan Hospital. Thirty right-handed MD outpatients between the ages of 40 and 60 years old (average age: 51.17 ± 6.06 years old), thirty right-handed MDD outpatients between the ages of 18 and 60 years old (average age: 37.50 ± 10.60 years old), and thirty right-handed healthy controls (HCs) between the ages of 18 and 60 years old (average age: 34.83 ± 8.77 years) were enrolled in this study. The subjects' demographic characteristics are presented in Table 1. A total of 20 participants used antidepressant medications including selective serotonin reuptake inhibitor (SSRI) therapy (*n* = 10) and SSRI with small doses of antipsychotic drugs (*n* = 10). The remaining female participants had no known history of treatment with antidepressants. Enrollment criteria for the MD patients included (a) a diagnosis of depressive disorder based on the Diagnostic and Statistical Manual of Mental Disorders fourth edition (DSM-IV) criteria; (b) an age between 40 and 60 years old, with an intact uterus and ovaries; the menopausal staging was determined using the Staging of Reproductive Aging Workshop + 10 stages; (c) a persistent difference of 7 days or more in the length of the menstrual cycle, an interval of amenorrhea of 60 days or longer, or stepping into the postmenopausal stage (at least 1 year after her final menstrual period) [25, 26]; (d) having a current psychiatric provider; (e) the presence of menopausal symptoms like vasomotor symptoms (hot flashes and night sweats), which are associated with hormonal fluctuations; and (f) the absence of hormonal replacement therapy.

Enrollment criteria for the MDD patients included (a) a diagnosis of depressive disorder based on the DSM-IV criteria; (b) all patients being females in order to eliminate the effect of gender factor; (c) being within the age range of 18–60 years old with an intact uterus and ovaries; (d) no vasomotor symptoms (hot flashes or night sweats) observed; and (f) the absence of hormonal replacement therapy.

All patients in the MD and MDD groups with other psychiatric disorders, chronic substance abuse, or severe medical illness related to cognitive function were excluded from the study. Enrollment criteria for the healthy control subjects included (a) the absence of major depressive disorder and other psychiatric disorders; (b) female subjects; (c) being within the age range of 18–60 years old with an intact uterus and ovaries; (d) the absence of obvious vasomotor symptoms (hot flashes and night sweats). All participants had an educational level of middle school or above. All patients in the MD and MDD groups were rated on the Hamilton Depression Rating Scale (HAMD, 24-item, Hamilton, 1960).

### 2.3. Assessment

All subjects were examined by two experienced psychiatrists according to the Structured Clinical Interview for DSM Disorders. The patients completed the 24-item HAMD questionnaire to determine the severity of depression. Furthermore, demographic information was collected using questionnaires developed by our group.

### 2.4. NIRS and VFT

**Figure 1:**
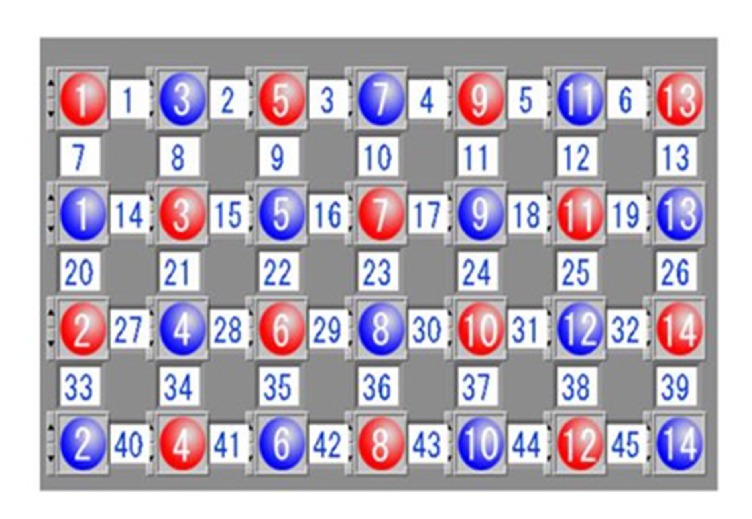
The area between 14 pairs of probes comprised 45 channels, 3 cm apart. The red color represents emission probes, and the blue color represents detector probes.

**Figure 2:**
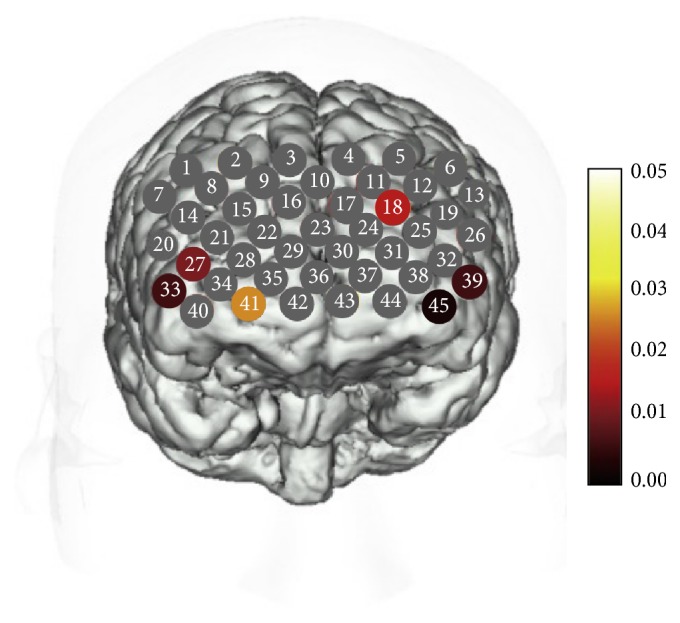
Change in the oxy-Hb levels among the three examined groups during the verbal fluency task. A significant statistical alteration in Oxy-Hb among the three examined groups was observed in six channels including three left channels (18, 39, and 45; *F* = 4.364, 5.282, and 6.732, resp.; *P* = 0.016, 0.007, and 0.002, resp.; FDR-corrected *P* = 0.027, 0.013, and 0.009, resp.) and three right channels (27, 33, and 41; *F* = 4.764, 5.062, and 3.702, resp.; *P* = 0.011, 0.008, and 0.029, resp., FDR-corrected *P* = 0.022, 0.018, and 0.04, resp.). The *P* values in the grey channels are nonhomogeneous and >0.029, whereas the *P* values in the colored channels are ≤0.029.

A 45-channel NIRS system (FOIRE-3000, Shimadzu Corporation, Japan) was used to measure the relative oxygenation changes of Oxy-Hb, HHb, and total hemoglobin (total-Hb) during the activation task. The relative values were calculated using the Lambert–Beer's law [20]. A total of 28 probes were placed on the forehead of each patient, including 14 emissions and 14 detectors, 3.0 cm apart (Figure 1). The area between the emission and the detector probes is the location of a channel. The measurement area contains the majority of the PFC (Figure 2). The lowest probes were positioned along the Fp1-Fp2 line according to the International 10-20 system of electroencephalogram electrode placement [27].

The activation task was comprised of four different semantic category versions of the VFT. VFT is a verbal task that aids in measuring cognitive functions. It is usually used in neuropsychological assessment of mental diseases [28]. It contains four blocks, that is, vegetables, family applications, four-footed animals, and fruits. Each of the blocks contained a 30-s pretask baseline, a 30-s task, and a 30-s posttask baseline. The cues were determined according to previously published research [29]. The semantic cues were visually presented by E-prime 2.0 software on a computer screen during measurement. The relative concentrations of Oxy-Hb, HHb, and total-Hb were measured between the rest period and the task period. The time resolution was set at 0.2 s. Baseline corrections and filtered data were used before data export. The subjects were asked to pronounce the names of as many items as possible within the semantic category. The number of correct words was recorded as the evaluation of cognitive function during the task period.

### 2.5. Statistical Analysis

Homogeneity of variance was used to compare the distribution shape in the three groups. One-way analysis of variance (ANOVA) was used to analyze the demographic characteristics (age and education level), clinical data (disease duration and HAMD score), and the number of correct items during the VFT among the three groups. The least significant difference (LSD) test was used to compare two of the three groups. The changes in Oxy-Hb and HHb were employed to evaluate the brain function, relying on the fact that the cerebral blood flow changes during VFT using NIRS. In order to calculate the changes of the Oxy-Hb and HHb levels, we subtracted the mean relative concentration of Oxy-Hb and HHb in the task period from that in the pretask period, respectively. In addition, we analyzed the differences in the Oxy-Hb and HHb concentration, respectively, among the three groups by covariance analysis (ANCOVA) with two correlated variables to avoid the effects of age and the severity of depression. We adopted the Benjamini-Hochberg procedure with the false discovery rate (FDR = 0.2) for the multiple 45-channel testing correction. For the calculation of Oxy-Hb, channels presenting *P* values ≤ 0.029 were considered to be statistically significant. For HHb, channels presenting *P* values < 0.004 were considered to be statistically significant. The LSD was used to process the post hoc test. The differences between parameters were analyzed by the Statistical Package for Social Sciences (SPSS, IBM Corporation, Armonk, NY, USA), version 18.0 in Windows.

## 3. Results

### 3.1. Analysis of Demographic Features and Clinical Characteristics

There were no significant differences in disease duration or education level among the examined groups. However, the ages between patients in the MD (average age: 51.17 ± 6.06 years old) and MDD (average age: 37.50 ± 10.60 years old) groups as well as the ages between patients in the MD (average age: 51.17 ± 6.06 years old) and HC (average age: 34.83 ± 8.77 years old) groups were significantly different (*P* < 0.05). We employed the HAMD scores to determine the severity of depression in the MD and MDD patients. We observed significant differences in the HAMD scores between patients in the MD and HC groups (*P* < 0.05) as well as between patients in the MDD and HC groups (*P* < 0.05).

### 3.2. Completion of the VFT

The results indicated the presence of statistically significant differences among the three groups in the family application, four-footed animal, and fruit blocks (Table 1). However, no significant difference was observed in the vegetable block. The differences between patients in the MD and HC groups were statistically significant in the family application, four-footed animal, and fruit blocks (*P* = 0.003, *P* = 0.001, and *P* = 0.001, resp.). The MDD group and the HCs were significantly different in the family application and four-footed animal blocks (*P* = 0.007, *P* = 0.036, resp.). None of the four categories were statistically significant between the MD and MDD groups (*P* > 0.05).

### 3.3. NIRS Data Analysis

We compared the alteration in Oxy-Hb concentration among the three groups via ANCOVA. Changes in the Oxy-Hb concentration were homogeneous in 29 channels (*P* > 0.05) but not in the following channels: 1, 2, 7, 14, 26, 28, 29, 31, 32, 34, 35, 36, 37, 38, 43, and 44 (*P* < 0.05). The FDR was adopted for multiple 45-channel testing correction. There were no significant interaction differences between the age factor and the group factor in seven channels (18, 27, 33, 39, 40, 41, and 45, *P* > 0.05). Significant interactions between the HAMD score factor and the group factor were identified in channel 40 (*P* < 0.05).

**Figure 3:**
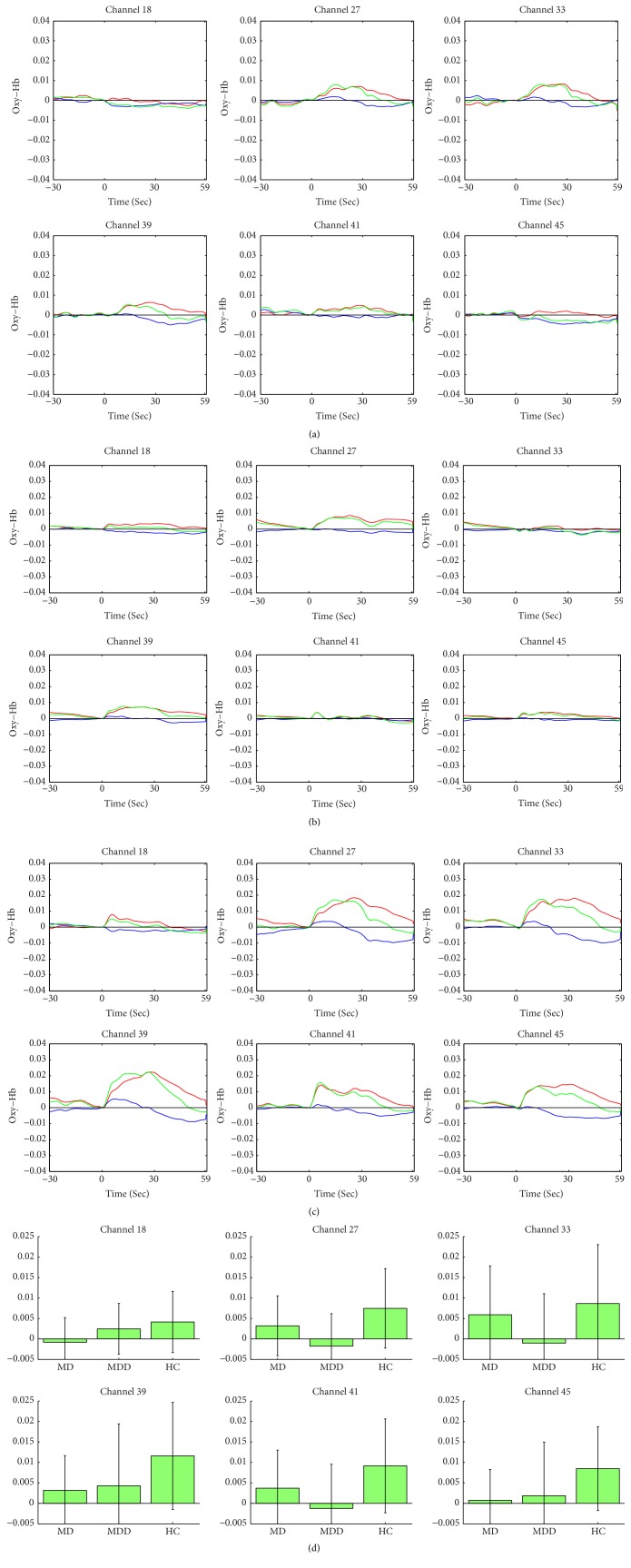
(a) Average waveforms of oxy-Hb (red line), deoxy-Hb (blue line), and total-Hb (green line) concentrations during the verbal fluency task in the MD group in 6 channels with near-infrared spectroscopy (NIRS) with respect to the time course, Hb: hemoglobin. (b) Average waveforms of oxy-Hb (red line), deoxy-Hb (blue line), and total-Hb (green line) concentrations during the verbal fluency task in the MDD group in 6 channels with NIRS with respect to the time course. Hb: hemoglobin, NIRS: near-infrared spectroscopy. (c) Average waveforms of oxy-Hb (red line), deoxy-Hb (blue line), and total-Hb (green line) concentrations during the verbal fluency task in the HC group in 6 channels with NIRS with the respect to the time course. Hb: hemoglobin, NIRS: near-infrared spectroscopy. (d) Histogram presenting the average alterations in Oxy-Hb levels between the task and pretask periods in six channels. Oxy-Hb: oxygenated hemoglobin.

We observed a significant statistical alteration in Oxy-Hb among the three examined groups in six channels including three left channels (18, 39, and 45; *F* = 4.364, 5.282, and 6.732, resp.; *P* = 0.016, 0.007, and 0.002, resp.; FDR-corrected *P* = 0.027, 0.013, and 0.009, resp.) and three right channels (27, 33, and 41; *F* = 4.764, 5.062, and 3.702, resp.; *P* = 0.011, 0.008, and 0.029, resp.; and FDR-corrected *P* = 0.022, 0.018, 0.04, resp.; Figure 3) The averaged waveforms of [oxy-Hb], [deoxy-Hb], and [total-Hb] during the verbal fluency cognitive task in the three groups (MD, MDD, and HC) are presented in Figures 3(a)–3(c).

The LSD test was used to examine the post hoc analysis of covariance. In the left dorsolateral PFC (DLPFC), we noticed a significant difference in channel 18 (*F* = 7.703, *P* = 0.007) between patients in the MD and MDD groups. Patients within the MD group displayed a significantly lower increase in the Oxy-Hb level compared to patients in the MDD group during the VFT (Figure 3(d)). Furthermore, we observed statistically significant differences in channels 18, 39, 41, and 45 (*F* = 5.542, 9.858, 4.030, and 13.326, resp.; *P* = 0.021, 0.002, 0.048, and <0.001, resp.) between patients of the MD and HC groups. The MD group showed a lower increased alteration in the Oxy-Hb levels compared to participants from the HC group during the VFT (Figure 3(d)).

Moreover, we observed a statistical difference in channels 27, 33, 39, 41, and 45 (*F* = 8.118, 9.475, 8.228, 7.402, and 8.543, resp.; *P* = 0.005, 0.003, 0.005, 0.008, and 0.004, resp.) between the MDD and HC groups. Patients in the MDD group presented a lower increase of the Oxy-Hb level compared to participants in the HC group during the VFT (Figure 3(d)). However, there was no significant difference between the MD group and the MDD group in channels 27, 33, 39, 41, and 45 (*F* = 2.807, 1.795, 0.987, 0.209, and 2.766, resp.; *P* = 0.098, 0.184, 0.323, 0.649, and 0.100, resp.). Similarly, there was no significant difference between the MD and HC groups in channels 27 and 33 (*F* = 0.253 and 1.619, resp.; *P* = 0.207 and 0.093, resp.), and no significant difference was observed between participants in the MDD and HC groups in channel 18 (*F* = 2.893 and *P* = 0.616).

Changes in the HHb concentration were homogeneous in 36 channels (*P* > 0.05) but not in the following channels: 1, 2, 7, 22, 24, 28, 30, 31, and 38 (*P* < 0.05). Significant interaction differences were observed between the age factor and the group factor in three channels (5, 26, and 32, *P* < 0.05). Significant interactions between the HAMD score factor and the group factor were identified in two channels (34 and 41, *P* < 0.05). The FDR was adopted for multiple 45-channel testing correction. Channels presenting *P* values < 0.004 were considered to be statistically significant. We did not observe significant alterations in the HHb levels among participants in the MD, MDD, and HC groups in the remaining channels (3, 4, 6, 8, 9, 10, 11, 12, 13, 14, 15, 16, 17, 18, 19, 20, 21, 23, 25, 27, 29, 33, 35, 36, 37, 39, 40, 42, 43, 44, and 45; *F* = 0.037, 1.614, 1.705, 0.344, 0.019, 0.984, 0.739, 3.407, 4.506, 0.023, 0.142, 0.476, 0.785, 1.028, 3.465, 2.071, 0.046, 1.853, 2.187, 0.413, 5.954, 1.259, 1.446, 0.799, 4.235, 0.919, 0.325, 1.191, 0.725, 0.649, and 4.174, resp.; FDR-corrected *P* = 0.187, 0.080, 0.076, 0.164, 0.196, 0.107, 0.133, 0.031, 0.009, 0.191, 0.178, 0.151, 0.129, 0.102, 0.027, 0.053, 0.182, 0.071, 0.044, 0.156, 0.004, 0.093, 0.084, 0.124, 0.013, 0.111, 0.169, 0.098, 0.138, 0.147, and 0.018, resp.).

## 4. Discussion

In this study, we used NIRS to investigate the Oxy-Hb alteration of PFC among female participants in the MD, MDD, and HC groups during VFT. The results of the VFT demonstrated that words generated during the family application, four-footed animal, and fruit blocks were significantly decreased in the MD and MDD patients, respectively, compared to the HC participants. Patients in the MD and MDD groups had a lower frontal lobe function than the HCs during the VFT performance. These findings were in agreement with those of Herrmann et al., who determined that the ability to find words based on a common criterion was primarily related to the frontal lobe function [30]. However, there was no difference between the MD and MDD groups in the presentation of words generated during the VFT. The number of generated words was similar between the MD and MDD groups. The exact cause for this phenomenon is not clear. Nevertheless, we observed different degrees of PFC activation during the VFT using NIRS.

NIRS is a noninvasive spectroscopic technique that has been used to measure brain function in several psychiatric disorders [21]. We focused on examining the Oxy-Hb concentrations during the 30-s task period. Hoshi et al. previously suggested that Oxy-Hb is the most sensitive indicator for activation studies using NIRS [31]. In a study using NIRS to monitor the change of Oxy-Hb levels, Matsuo et al. observed that hypofrontality in mood disorders was associated with a poorer response of blood vessels to stimuli [22]. Furthermore, using the frontal hemodynamic patterns detected by NIRS, Takizawa et al. reported a significant difference in the PFC between MDD patients and patients with bipolar disorder or schizophrenia accompanied by symptoms of depression [23]. In addition, results obtained by Matsubara et al. indicated abnormal PFC activation in MDD and bipolar disorder patients with NIRS [24]. Additionally, the fundamental role of PFC in depression has been reported by previous studies by Takizawa et al. and Matsubara et al. [23, 24]. Therefore, in this study, we examined the difference in PFC activation among female patients presenting with MD or MDD with NIRS. To the best of our knowledge, this is the first study to measure the change of Oxy-Hb during VFT among MD, MDD, and HC groups with NIRS.

In the areas of channels 18, 27, 33, 39, 41, and 45, the three examined groups exhibited significantly different degrees of activation during the VFT in the bilateral DLPFC. We observed a lower activation in both the left and right DLPFC during the course of task segments in the MD patients (channels 18, 39, 41, and 45) and the MDD patients (channels 27, 33, 39, 41, and 45), compared with the HCs. The reduction in Oxy-Hb activation during the VFT period implies that patients in the MD and MDD groups might have difficulty in acquiring adequate blood supply to compensate for the consumed oxygen. This compensation mechanism is crucial for proper neuronal activity. The area of altered Oxy-Hb concentration was mainly located in the bilateral DLPFC, which is known to play a key role in cognition [22–24]. In good agreement with previous reports, the results obtained from this study confirm that the hemodynamic hypoactivation in the DLPFC is associated with depression [32, 33].

Moreover, compared to the HC group, the patients in the MDD group presented with a lower activation in the bilateral DLPFC in channels 27, 33, 41, 39, and 45 (left DLPFC and right DLPFC) (Figure 3(d)). These results are in accordance with previous reports indicating that the abnormal PFC functions were mood-dependent [34, 35]. Additionally, Kinou et al. have previously shown a lower-than-normal Oxy-Hb activation during the VFT in the dorsolateral and ventrolateral PFC in patients with MDD [36].

Furthermore, a lower activation in these four channels (channels 18, 39, 41, and 45) of the bilateral DLPFC was observed in the MD group, compared to the HC group. However, a previous study by Saletu et al. has indicated that MD is correlated to right frontal hyperactivation and left frontal hypoactivation using electroencephalography [37, 38]. Our results showed hypoactivation of both the left and right DLPFC. Moreover, in this study, in channel 18 (Brodmann area 46), we observed hypoactivation of the DLPFC in the MD group when compared to the MDD group. The results showed that the MD group exhibited a lower Oxy-Hb activation in channel 18 (Brodmann area 46) of the left DLPFC compared to that of the MDD group. In addition, Fox et al. have reported previously that the symptoms of depression were improved through stimulating Brodmann area 46 with transcranial magnetic stimulation, compared to stimulating other areas of the brain cortex [39]. This finding indicates that Brodmann area 46 is usually correlated to depressive disorders. During the menopausal period, females often experience a variety of symptoms, like vasomotor symptoms, which can be attributed to hormonal fluctuations. Patients presenting with MD usually experience depression and hormonal fluctuations [3, 7]. In this study, the Oxy-Hb hypoactivation in channel 18 (Brodmann area 46) during the VFT period suggests that the repeated loss of overcompensation for the blood supply could result in a shortage of reserved energy in the PFC; in addition, it leads to neurological side effects possibly due to hormonal fluctuations. Therefore, the NIRS results indicate that channel 18 (Brodmann area 46) in the left DLPFC may be a biomarker in MD patients.

## 5. Limitations

This is a retrospective study; therefore, the difference in age among the examined groups is a main limitation of the study. In order to reduce the impact of the age difference on our results, we used ANCOVA. Nevertheless, future studies should include age-matched female participants. Furthermore, we need to investigate the effect of medications administered and hormonal fluctuations. Therefore, future studies including more menopausal patients that correlate hormonal fluctuations to the severity of depression symptoms and Oxy-Hb alterations will be necessary to confirm the results obtained here.

## 6. Conclusion

In conclusion, this study provides novel evidence that Brodmann area 46 may be a unique functional area that identifies and differentiates MD from MDD. Patients in the MD group showed an independent dysfunctional area in the left DLPFC (channel 18) that differed from the MDD group in terms of the hemodynamics. These findings may represent a novel pathophysiology for MD patients and may provide insights regarding its treatment.

## Figures and Tables

**Table 1:** Demographic and clinical data as well as the number of items generated during the VFT from the MD and MDD patients and HCs.

Demographics	MD	MDD	HC	ANOVA	MD versus MDD	MD versus HC	MDD versus HC
				*P*	*P*	*P*	*P*
Gender (female/male)	30/0	30/0	30/0				
Age (years)	51.17 ± 6.06	37.50 ± 10.60	34.83 ± 8.77	<0.001	<0.001	<0.001	0.237
Disease duration (years)	3.23 ± 3.87	1.70 ± 2.83	—	0.085	—	—	—
Education level (years)	13.43 ± 2.46	14.57 ± 2.73	14.73 ± 1.78	0.072	—	—	—
Medication (with/without)	13/17	7/23	—	—	—	—	—
HAMD	23.23 ± 7.68	22.93 ± 10.16	3.96 ± 2.50	<0.001	0.877	<0.001	<0.001
Vegetables	9.63 ± 3.89	11.17 ± 4.05	11.07 ± 3.57	0.230	0.126	0.152	0.920
Family applications	8.27 ± 3.04	8.53 ± 3.07	10.57 ± 2.45	0.004	0.719	0.003	0.007
Four-footed animals	7.97 ± 2.60	8.83 ± 2.53	10.30 ± 2.87	0.004	0.212	0.001	0.036
Fruit	8.53 ± 3.07	10.07 ± 3.72	11.43 ± 3.17	0.005	0.078	0.001	0.116

MD: menopausal depression, MDD: major depressive disorder, HC: healthy control.

## Abbreviations

ANOVA: One-way analysis of variance
ANCOVA: Covariance analysis
DLPFC: Dorsolateral prefrontal cortex
DSM-IV: Diagnostic and Statistical Manual of Mental Disorders, fourth edition
FDR: False discovery rate
HAMD: Hamilton Depression Rating Scale
HC: Healthy control
HHb: Deoxygenated hemoglobin
LSD: Least significant difference
MD: Menopausal depression
MDD: Major depressive disorder
NIRS: Near-infrared reflectance spectroscopy
Oxy-Hb: Oxygenated hemoglobin
PFC: Prefrontal cortex
SSRI: Selective serotonin reuptake inhibitors
Total-Hb: Total hemoglobin
VFT: Verbal fluency task.
